# Strategies to Combat Caries by Maintaining the Integrity of Biofilm and Homeostasis during the Rapid Phase of Supragingival Plaque Formation

**DOI:** 10.3390/antibiotics11070880

**Published:** 2022-06-30

**Authors:** Paola Hernández, María C. Sánchez, Arancha Llama-Palacios, María J. Ciudad, Luis Collado

**Affiliations:** 1 Department of Medicine, Faculty of Medicine, University Complutense, 28040 Madrid, Spain; leidypah@ucm.es (P.H.); mariasan@ucm.es (M.C.S.); mallamap@ucm.es (A.L.-P.); lcollado@ucm.es (L.C.); 2 GINTRAMIS Research Group (Translational Research Group on Microbiota and Health), Faculty of Medicine, University Complutense, 28040 Madrid, Spain

**Keywords:** oral biofilm, dental plaque, dental caries, homeostasis, dysbiosis, anticaries therapeutic approaches

## Abstract

Bacteria in the oral cavity, including commensals and opportunistic pathogens, are organized into highly specialized sessile communities, coexisting in homeostasis with the host under healthy conditions. A dysbiotic environment during biofilm evolution, however, allows opportunistic pathogens to become the dominant species at caries-affected sites at the expense of health-associated taxa. Combining tooth brushing with dentifrices or rinses combat the onset of caries by partially removes plaque, but resulting in the biofilm remaining in an immature state with undesirables’ consequences on homeostasis and oral ecosystem. This leads to the need for therapeutic pathways that focus on preserving balance in the oral microbiota and applying strategies to combat caries by maintaining biofilm integrity and homeostasis during the rapid phase of supragingival plaque formation. Adhesion, nutrition, and communication are fundamental in this phase in which the bacteria that have survived these adverse conditions rebuild and reorganize the biofilm, and are considered targets for designing preventive strategies to guide the biofilm towards a composition compatible with health. The present review summarizes the most important advances and future prospects for therapies based on the maintenance of biofilm integrity and homeostasis as a preventive measure of dysbiosis focused on these three key factors during the rapid phase of plaque formation.

## 1. Introduction

Dental caries is considered one of the most prevalent human diseases, with the 2019 Global Burden of Disease Study estimating that close to 3.5 billion people worldwide experience oral disease, with caries in permanent teeth being the most common condition [1]. Globally, it is estimated that 2 billion people are affected by caries in permanent teeth and that 520 million children are affected by caries in primary teeth [1].

The main ethological component of dental caries consists of bacterial species (mainly streptococci, lactobacilli, and bifidobacteria) organized in sessile and highly specialized communities (the supragingival dental plaque) located in the proximal spaces and occlusal surfaces of the gingival margin [2,3,4,5,6,7]. Advances in the various omics techniques applied to the field of dental plaque have revealed that this oral ecosystem is inhabited by hundreds of bacterial species, most of which are considered commensal but include low levels of opportunistic pathogens that become the dominant species in caries-affected sites at the expense of health-associated taxa [8,9,10,11,12,13,14,15,16].

The conformation of the supragingival biofilm can be influenced by a wide range of factors, including the intake of food and drink, the availability of endogenous nutrients, drug treatments, the host’s immune system, and systemic diseases [17,18,19,20]. To respond to these factors, the oral microbiota maintains the internal stability of the community through homeostasis. However, certain ecological changes can induce modifications in the oral microbiota, leading to dysbiosis and dental caries [20,21,22,23,24]. Saliva plays an important role in oral microbial ecology by supplying nutrients and providing protection against colonization by nonoral organisms. However, reduced salivary flow has a major effect on the microbiota, resulting in increased growth/colonization by opportunistic pathogens, including non-oral bacteria and fungi. Certain systemic diseases, such as diabetes, also affect the oral microbiome, raising glucose levels in saliva and tissue and affecting bacterial nutrition. Similarly, excessive and/or frequent consumption of fermentable carbohydrates affects the composition of the oral bacterial community. The changes induced in these ecological ecosystems by the aforementioned factors are directly related to bacterial biofilm dysbiosis and the onset and progression of dental tissue damage [19,20]. If the microbiota changes, there will be a proliferation of aciduric microorganisms, mainly represented by *Streptococcus*, *Lactobacillus*, *Bifidobacterium*, *Actinomyces*, *Veillonella*, and certain yeasts, among others. Subsequently, the oral pH falls below the critical point for the demineralization process for an increased period, resulting in dental tissue demineralization [20,21,23,24].

The biofilm phenotype of oral-caries-causing bacteria provides them with substantial resistance to anticaries therapies. Thus, for example, mechanical oral hygiene can limit microbial growth but does not completely eliminate the biofilm, which recomposes itself in a few hours [23,25,26]. Pharmacological treatments based on antibiotic and antiseptic therapy are less effective against bacteria organized in biofilm than against planktonic phenotypes, not exempt, in addition, to unwanted effects [18,26,27]. Despite the knowledge generated about the onset and evolution of bacterial dysbiosis as a caries-causing agent, there are few therapies focused on combining more conventional routine mechanical treatment, such as tooth brushing or rinsing assisted by antiseptic compounds to control the natural growth of dental plaque, with newer methods that act on the remaining percentage of plaque, which has survived and will regenerate in a few hours, helping to maintain homeostatic capacity.

In this review of the literature, we focus on describing state-of-the-art methods showing the most important advances and future prospects for this type of therapy based on the maintenance of biofilm integrity and homeostasis as a preventive measure of dysbiosis. 

## 2. From Homeostatic to Dysbiotic Supragingival Biofilm

The mouth is a warm and humid habitat that exposes numerous surfaces, including the mucosa, which are covered by keratinized and nonkeratinized stratified squamous epithelium, the papillary surface of the tongue dorsum or the hard structures of the teeth, which lie above (supragingival) and below (subgingival) the gingival margin, all of them susceptible to colonization by a wide variety of microorganisms. Teeth have a unique characteristic in the human body, providing major advantages for colonization by microorganisms: a nonscaling rigid surface exposed to an environment abundant in nutrients, facilitating microorganisms’ organization into sessile communities [2,4,5]. Despite its advantages, this region also presents nonfavorable conditions for microorganisms, which induce bacteria to aggregate into biofilms and thereby survive in the extreme conditions in a continuously changing environment caused by changes in fluids such as saliva and crevicular fluid if they access the subgingival zone, oxygen concentration gradients, environmental stress, invasion by other competing microorganisms, and the unbalanced availability of nutrients derived from eating and chewing [5,17,28].

**Figure 1:**
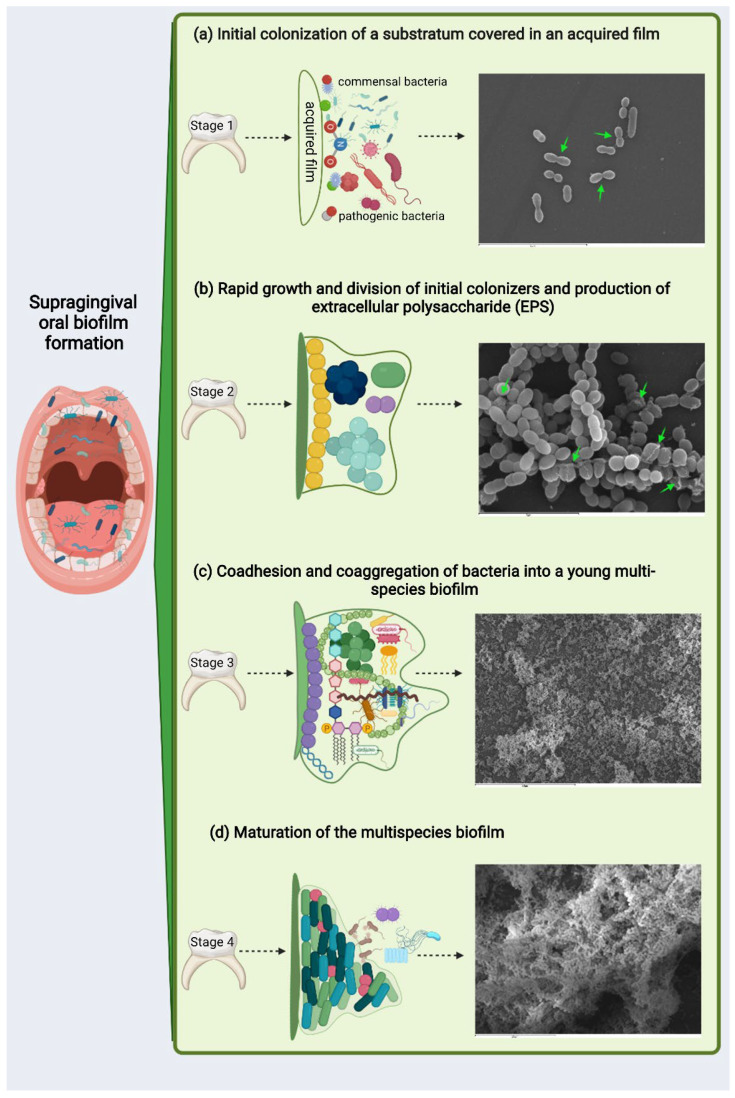
Illustration exemplifying the possible development of multispecies biofilms. Scanning electron microscopy corresponds to an in vitro supragingival biofilm model of (**a**) 3, (**b**) 12, (**c**) 24, and (**d**) 48 h of evolution (to methodology [9]): (**a**) initial colonization of a substratum covered in an acquired film composed of polysaccharides and proteins. In vitro, various bacteria of coccoid and bacillary morphology can be seen on the surface after 3 h of biofilm process initiation; cell division can be appreciated mainly in coccoid bacteria (green arrows; scale bar = 9 µm); (**b**) rapid growth and division of initial colonizers and production of extracellular polysaccharide (EPS) leading to the development of microcolonies from several bacterial species—green arrows point to EPS surrounding bacteria in a microcolony after 12 h of biofilm development in vitro, which is identifiable in the image as a compact mass of greater brightness—(scale bar = 5 µm); (**c**) coadhesion and coaggregation of bacteria into a young multispecies biofilm—after 24 h of in vitro evolution, the surface appeared to be covered by bacteria consisting primarily of a larger colonies—outward-growing masses of bacterial cells alternating with flat homogenous layers of cells (scale bar = 50 µm); and (**d**) maturation of the multispecies biofilm—after 48 h of in vitro incubation, biofilm demonstrated the characteristic organization of these communities: covering the surface with bacteria clusters, forming stacks, and showing channels inside the structure—(scale bar = 20 µm).

Supragingival biofilm formation occurs within a few hours in a series of sequential but arguably almost simultaneous events (Figure 1). The normal development of dental plaque starts with the selective adsorption of hydrophobic macromolecules onto the tooth surface —constantly bathed in saliva rich in glycoproteins and other proteins— to create a conditioning film. The glycoproteins and proteins in the conditioning film serve as ligands, attracting specific species of Gram-positive and Gram-negative bacteria mainly from the genera *Streptococcus*, *Actinomyces*, *Capnocytophaga*, *Eikenella*, *Haemophilus*, *Prevotella*, *Propionibacterium*, and *Veillonella* (Figure 1a) [28,29,30].

Once the surface has been successfully colonized by these pioneer bacteria, the biofilm has already begun to form and will start differentiating as a highly structured community. To do this, the bacteria (and, consequently, the biofilm) enter a logarithmic growth phase (Figure 1b). During this intermediate phase, there is faster growth in the evolution of the biofilm of Gram-positive bacteria compared to Gram-negative species. Interspecies communication, now mediated by the quorum-sensing (QS) system, will facilitate biofilm differentiation, influencing bacterial metabolism and leading to extracellular polysaccharide matrix formation (EPS) and community maturation [28,29,31]. 

The union and growth of the first colonizers on the dental surface provides new ligands for colonization by other species that adhere successively, mainly from the genera *Corynebacterium*, *Eubacterium*, and *Fusobacterium*, among others [30,32,33,34]. At this stage, a high bacterial density will have been reached, which entails significant oxygen consumption by the bacteria, thereby generating oxygen gradients inside the biofilm structure (Figure 1c). An anaerobic environment then begins to develop, which will favor the growth of these secondary colonizers, which have stricter anaerobic requirements for their growth. Anaerobic taxa tend to be in the interior, whereas facultative or obligate aerobes are located at the periphery of the consortium. Consumers and producers of certain metabolites, such as lactate, tend to be near each other [33,34,35]. Such highly organized spatial arrangements are likely to result from and facilitate a large variety of interspecies interactions, including the formation of metabolic networks [27,34,35].

Synergistic and antagonistic interactions with neighboring species will be established at this time in the biofilm, generating an exchange of food and oxygen that strengthens the biofilm matrix and creating the ideal environment for the coexistence of bacteria with diverse survival mechanisms. The biofilm thereby reaches a mature state and equilibrated homeostasis (Figure 1d) [30,32]. Synergism includes the collective degradation of salivary glycoproteins by microbial consortia, in which complementary enzymatic activities allow the utilization of mucins in saliva as an energy source or of food chains in which a metabolic product of one species is used as the primary energy source for a partner species. Antagonistic interactions are mediated by the production of bacteriocins and hydrogen peroxide (H_2_O_2_) [36,37,38]. When there is a balance between acid production and alkaline compensation, the biofilm enters into symbiosis with the host, establishing a neutral pH between the biofilm and the ecosystem. This condition allows the biofilm to be stable in its bacterial composition and helps regulate and strengthen the inflammatory response provided by the host’s immune cells to attack by pathogenic bacteria. At this mature phase, the biofilm and host can coexist in harmony [27,39].

The last step in the development of a biofilm is the release and migration of bacteria to other surfaces, which causes a reorganization of the microbial structure and composition of the biofilm [3,30,32].

Normal supragingival biofilm development can, however, be affected by the various aforementioned factors, which the bacterial community will be unable to counteract by the homeostatic capacity, and, as a result of the ecological changes, the microbiota will be modified. An increase in carbohydrates in the environment to be metabolized by bacteria and the subsequent generation of a low environmental pH can change the composition and metabolic properties of the bacterial communities in dental plaque, leading to enrichment of acid producers (acidogenic) and acid-tolerant (aciduric) microorganisms [21,40]. The overgrowth of these bacteria and the excessive carbohydrate fermentation modify the oxygen gradient, creating a more anaerobic environment, thereby favoring rapid growth of pathogenic genera such as *Porphyromonas*, *Tannerella*, *Treponema*, *Capnocytophaga* and *Aggregatibacter*, which were present in very low latent numbers and were waiting for favorable conditions to grow in the biofilm [35,40]. In this dysbiotic condition, the supragingival biofilm could be the initial point for developing diseases other than caries, such as periodontal and peri-implant diseases [21,38].

## 3. Benefits Derived from Biofilms in Homeostasis and the Disadvantages of Dysbiosis

The supragingival biofilm in homeostasis promotes oral health, providing advantages to the bacterial community and the host. In contrast, its dysbiosis can cause certain deleterious effects on both.

Resident commensal microbiota behave as a barrier against invasion by other competing microorganisms, preventing the colonization of this niche. Streptococci are the main early colonizers due to their ability to bind to any cell, whether human or bacterial [41]. Even at the onset of biofilm formation, species such as *Streptococcus mitis*, *Streptococcus sanguinis*, and *Streptococcus cristatus* use arginine deiminase to suppress the expression and production of fimbrial proteins and gingipains in pathogenic bacteria such as *Porphyromonas gingivalis* and reduce their adhesion [42,43,44].

The buffer capacity of saliva also benefits from commensal microbiota. Species such as *Streptococcus salivarius*, *S. mitis*, *Streptococcus gordonii*, *S. sanguinis*, *S. cristatus*, and *Actinomyces naeslundii* buffer the acid produced by certain cariogenic bacteria through the salivary lactoperoxidase system, which increases H_2_O_2_ and the pH of saliva. This action favors the saliva’s ability to neutralize acid and maintain the demineralization–remineralization process [36,45,46].

Commensal bacteria in homeostasis, through antagonistic bacterial interactions mediated by the production of bacteriocins and H_2_O_2_, can also affect community association, as occurs with *S. mitis* and/or *Haemophilus parainfluenzae*, which enhance the behavior of other caries-related streptococci [47,48]. Commensal bacteria also help reduce nitrate, which is essential for homeostasis and cardiovascular health. Through the action of nitrate reductase enzymes, commensal facultative anaerobic bacteria located in the tongue can reduce nitrate to nitrite, which functions as a vasodilator and antihypertensive when it passes into the bloodstream [49,50].

Biofilm in homeostasis strengthens the immune response and provides important benefits to the host. Oral streptococci suppress cytokine expression in epithelial cells, which reduces the inflammation of epithelial cells in oral mucosa and stimulates type I and II interferon response [51,52,53]. Commensal *Lactococcus lactis* also produces an antibiotic substance called nisin, which helps reduce tumor formation, cancer cell migration, squamous cell invasion, and dental caries [54,55,56,57,58]. 

In contrast, dysbiosis represents the alteration of the symbiotic state of the oral microbiome on the supragingival surface of the teeth [20,38]. Most of the enzymatic activities of bacteria that allow them to adapt to an environment are influenced by the acidification of the environment. When there is a high and constant consumption of carbohydrates and fermentable sugars, the pH of the medium alters from an alkaline condition (6.0–7.0)—favorable for commensal bacteria—to a more acidic 5.5, which decreases the flow and buffering capacity of saliva [24,59], resulting in changes in the bacterial composition. These changes favor the rapid growth of *Streptococcus*, *Actinomyces*, and *Lactobacillus*, which metabolize carbohydrates more easily, degrading glucose into pyruvate, lactate, and acetate, generating an anaerobic environment [9,59]. In this scenario, the growth of certain species of *P. gingivalis*, *Treponema denticola*, *Tannerella forsythia*, and *Aggregatibacter actinomycetemcomitans* also produce other acids, such as lactic and formic acid [9,59,60], which complicate the situation. The accumulation of more diverse microbiota tolerant to an acidic environment on the supragingival surface of the tooth, along with elevated carbohydrate fermentation, disrupts the demineralization–remineralization balance of dental tissues, facilitating the onset of carious lesions [23,40,60,61].

The dysbiosis of supragingival biofilm also decreases the H_2_O_2_ production of *S. gordonii* and reduces its competitiveness against *Streptococcus mutans* [37,45]. Likewise, the dysbiosis promotes the growth of cariogenic bacteria, such as *S. mutans*, *Lactobacillus*, *Actinomyces*, and *Veillonella* [21,39,40]. These bacteria produce lactic acid, which alters the exopolysaccharide matrix and modifies the chemical gradients that contribute to decreasing the pH of the medium. These alterations affect the genotypic and phenotypic selection of the microorganisms within the biofilm consortium, increasing their cariogenicity and causing the demineralization of dental tissues. If left untreated, this can lead to more advanced oral pathologies, such as pulp involvement, that can lead to abscess formation [38,61].

## 4. Strategies to Combat Caries by Maintaining the Integrity of Biofilm and Homeostasis during the Rapid Phase of Supragingival Plaque Formation 

In view of the aforementioned, adequate methods of homeostatic maintenance of oral biofilms are currently a subject of research, as it could prevent the onset and progression of caries.

**Figure 2:**
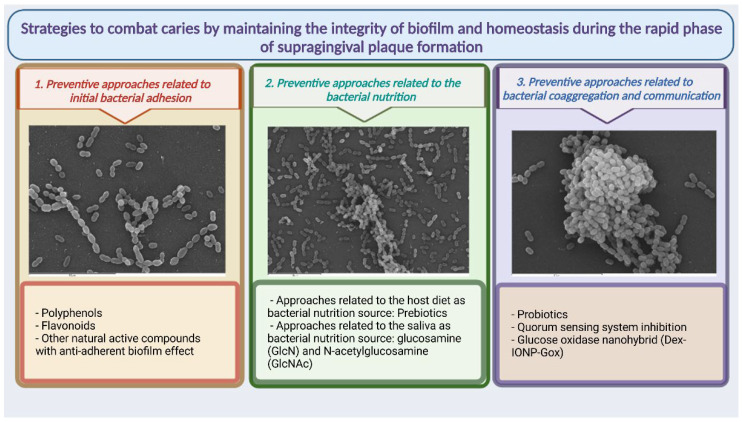
Summary of possible therapeutic approaches presented in the revision aimed at strategies to combat caries by maintaining the integrity of biofilm and homeostasis during the rapid phase of supragingival plaque formation.

Dental plaque is mainly affected by oral hygiene. To effectively control plaque, tooth brushing is combined with toothpaste or mouthwashes that contain a variety of chemical agents; however, this does not provide optimal plaque removal [62], an issue that has been extensively reviewed, resulting in the biofilm remaining in an immature state with high proportions of early-plaque-forming bacteria, particularly streptococci. In the rapid phase of supragingival plaque formation from firmly attached bacteria on a previously cleaned surface, bacteria double in number in 3–4 h [63]. Adhesion, nutrition, and communication are essential in this phase for bacteria that have survived those adverse conditions to rebuild and reorganize the biofilm. These three bacterial biofilm development factors can be the target of preventive strategies to guide the biofilm towards a composition compatible with health and equally healthy metabolic behavior (Figure 2). The preventive strategies at this phase mainly act on the primary colonizers—those that can quickly begin biofilm restoration. This review therefore summarizes the most important advances and future prospects for this type of therapeutic approach aimed at restoring the ecological balance at this immature state to prevent dysbiosis.

### 4.1. Preventive Approaches Related to Initial Bacterial Adhesion

The initial adhesion phase is a crucial step in designing strategies to intervene in the development of a cohesive and stable plaque structure compatible with health. However, this is a very ambitious project. Although various mechanisms have been described to inhibit specific bacterial adhesion and even complete biofilm attachment, the bacterial community as a whole has a wide variety of alternative strategies that allow it to circumvent such approaches. Several studies have presented compounds that by various means inhibit bacterial adhesion in vitro. This is the case for graft copolymer M239-144, which acts significantly on the hydrophobicity-mediated adhesion of *Streptococcus* species but does so less effectively in vivo [64,65]. Natural polyphenols and other active plant-derived compounds have also been studied. Current studies have focused on evaluating the ability of active plant-derived compounds to reduce the adhesion of pathogens and inhibit the formation of biofilms in disease development processes [66,67]. Phytochemicals, mainly polyphenols and flavonoids, have been widely studied to manage the growth inhibition, acid production, and adhesion of pathogens during biofilm formation and to prevent dysbiosis [68].

#### 4.1.1. Polyphenols

Various natural sources provide polyphenols with antimicrobial effectiveness. Green tea (*Camellia sinensis*) contains epigallocatechin gallate, a polyphenol with a strong antimicrobial effect capable of suppressing the acid production of *S. mutans* [68]. In addition, this polyphenol promotes the adhesion of other beneficial streptococci, controlling the efficacy and safety between the medium and oral homeostasis. Black tea maintains the pH of the medium and inhibits bacterial glycolysis in the supragingival biofilm [67].

Blueberries (*Vaccinium macrocarpon*) are also rich in polyphenols, such as proanthocyanidins, that prevent the adhesion of pathogens such as *Escherichia coli* and *Helicobacter pylori* to mucous membranes [69]. Yamanaka A. et al. [70] described the capacity of cranberry juice in preventing the progression of supragingival plaque to dental caries via the reduction in the activity of fructosyltransferase and glucosyltransferase enzymes in charge of glucose metabolism and the formation of the EPS matrix. Cranberry juice also inhibits the adhesion of *S. mutans* to hydroxyapatite. These inhibitory and antibacterial effects were also observed with other natural extracts, such as clove methanol and aqueous and clove methanol extracts of *Syzygium aromaticum* and Myrtaceae, according to Rahim Z. et al. [71]. In a study by Karygianni [72], *Pistacia lentiscus* and *Olea europaea* (oleuropein, maslinic acid, hydroxytyrosol, oleocanthal, oleacein) extracts also reduce the acidogenicity and metabolic activity of oral biofilms, affecting *P. gingivalis* and *Fusobacterium nucleatum*. Philip N et al. [73] and other authors observed that berries inhibited the growth of *S. mutans*.

#### 4.1.2. Flavonoids 

Numerous flavonoids have been analyzed; at the oral biofilm level, however, only a few have shown efficacy in maintaining homeostasis. Artocarpine and artocarpesin from *Artocarpus heterophyllus* have shown effectiveness in inhibiting the growth of oral cariogenic bacteria, such as *S. mutans* and certain *Actinomyces* and *Lactobacillus* species [68]. Other flavonoids with a high capacity to interfere with the uptake of glucose and other metabolites in *S. mutans* and other cariogenic bacteria are the phytoalexin flavones and the erycristagallin present in *Erythrina variegata* (Leguminosae) [68]. The therapeutic effect of baicalein (5,6,7-trihydroxyflavone), a flavonoid present in *Scutellaria baicalensis* and *Scutellaria lateriflora*, is being evaluated but is believed to affect the expression of several virulence genes that interact with the genes of *S. mutans*, which would alter the progression of biofilm to caries [74]. Propolis contains flavonoids which have conferred good antimicrobial activity against a number of oral bacteria and inhibition of the adherence of *S. mutans* and *Streptococcus sobrinus* [68].

#### 4.1.3. Other Natural Active Compounds with Anti-Adhesion Biofilm Effect 

Rhodiola (*Rhodiola rosea*) is a plant whose extract can inhibit *S. mutans* when combined with other extracts from, for example, *Psidium* sp., *Mangifera* sp. and *Mentha* sp. These inhibit the adhesion of *S. mutans* and prevent acid production that can change the medium’s pH [75,76]. When combined with *Psidium* sp., *Mangifera* sp., and *Mentha* sp. natural extracts, a reduction has been observed in the hydrophobicity of the cell surfaces of *S. sanguinis* and *S. mutans*, reducing adherence in biofilm formation. *Psidium* sp. extract has also been observed to inhibit adherence of these bacteria in the biofilm, while *Mangifera* sp. decreases the initial pH change in mixed populations of *S. sanguinis* and *S. mutans* [77].

Babchi (*Psoralea corylifolia*, Fabaceae) contains bakuchiol, which has shown antibacterial activity against Gram-positive and Gram-negative bacteria. In oral biofilms, bakuchiol inhibits the growth and adhesion of *S. mutans*. Another terpene with antimicrobial activity is Sagittaria A-D from the arrowhead plant (*Sagittaria sagittifolia*), which has shown activity against *S. mutans*, *A. naeslundii*, and *Actinomyces viscosus* [68]. 

Xylitol—a sugar alcohol found naturally in plants such as birch (Betula), bark plants, and fruits—has anticariogenic properties, inhibits *S. mutans*, *S. saliviarius*, and is widely included in toothpastes, mouthwashes, and foods to prevent tooth decay [78].

In addition to the natural active compounds already mentioned, other herbs described in different studies, such as bloodroot, chamomile, caraway, myrrh, echinacea, rosemary, sage, aloe vera, thyme, and other useful herbs in dentistry may be good alternatives to current treatments for oral health problems, but clearly, more research is required to study their effectiveness on caries by maintaining the integrity of biofilm and homeostasis at the rapid phase of supragingival plaque formation [79].

### 4.2. Preventive Approaches Related to Bacterial Nutrition

The primary nutritional source for oral bacteria at the supragingival level is saliva [28,30,32], although the host’s diet is used by the bacteria in dental plaque. Bacteria attached to the tooth surface use salivary glycoproteins and dietary carbohydrates (mainly by streptococci) and proteins, which are degraded mostly by anaerobic Gram-negative proteolytic bacteria and amino acids and consumed by bacteria with aminopeptidase activity [80].

#### 4.2.1. Preventive Approaches Related to Host Diet as Bacterial Nutrition Source

Scientific evidence has shown that an increased frequency of carbohydrate intake will favor the cariogenic process, but other properties, such as food texture and the amount of time food remains in the oral cavity, should be taken into account in the disease’s progression [28,81]. For example, the consumption of soft drinks and sugary beverages decreases the pH of dental plaque in vivo [81,82]. Acids are neutralized by saliva, and it is estimated that saliva can neutralize the pH of plaque 20–30 min after consumption. However, if the consumption of acids is repetitive, the saliva’s capacity decreases, and the time for the bacteria to act is extended, leaving the tooth surface susceptible to the development of caries. The same situation occurs with sugary and sticky foods that remain longer on the tooth surface, releasing acids slowly and constantly so that the bacteria continue to metabolize the acids and causing caries to progress [38,81,82]. 

Surprisingly, a specific group (the amino sugars) related to carbohydrates can be considered a strategy for caries control and prevention focused on homeostatic balance. This group of compounds includes sugar molecules that contain an amino group instead of a hydroxyl group in one of their radicals and those derivatives of amines that contain sugars, such as N-acetylglucosamine and sialic acid, which—although they formally do not contain primary amines—are also considered amino sugars [83,84]. It has been reported that amino sugars can enhance the beneficial properties of oral streptococci and can moderate the cariogenicity of oral biofilms without disturbing homeostatic balance [40]. Amino sugars such as glucosamine and N-acetylglucosamine can increase the competitiveness of *S. gordonii* against *S. mutans*, reducing the viability of *S. mutans* against other commensal species. Streptococci can also metabolize amino sugars and arginine deiminase more easily, increasing H_2_O_2_ production and releasing ammonia, which raises the medium’s pH [80,83,84]. 

#### 4.2.2. Preventive Approaches Related to Saliva as a Bacterial Nutrition Source 

In terms of saliva as the oral bacteria nutrition source, prebiotics play a relevant role in prevention and are defined as a substance or molecule that can be used by microorganisms and can alter bacterial growth or metabolic activity to confer benefits to the host. In the oral cavity, prebiotics help nonpathogenic bacteria balance the oral microbiome, showing promising results in combating the progression of biofilm dysbiosis [51,85]. In certain cases, these benefits for the host will not only include control of plaque homeostasis but will also have systemic repercussions.

Nitrate is being studied as a possible prebiotic to beneficially modify plaque microbiota and play a protective role in maintaining cardiovascular health [49,50]. Through the enterosalivary circuit, nitrate from the diet or taken as a prebiotic supplement is absorbed in the stomach and passes into the bloodstream to later return in a concentrated form in the saliva, where it is reduced to nitrite by oral bacteria to ultimately become nitric oxide [50,86]. Nitrate supplementation has been observed in vivo to induce certain changes in the microbiota, such as an increase in nitrate-reducing bacteria, including *Neisseria* and *Rothia*, which are obligate aerobic bacteria related to oral health [87]. Nitrate supplementation has also been shown to have significant effects in lowering blood pressure [49,50,86].

Due to its ability to act during the initial phase of biofilm development to the detriment of the progression of dysbiosis towards caries, the amino acid arginine (L-arg) is also postulated as a good prebiotic in the oral cavity [44,80]. L-arg, present in peptides and proteins of salivary secretions [88], is metabolized by the arginine deiminase system (ADS) to release ornithine, ammonia, and carbon dioxide to counteract the acid production of the medium, protect cells from acidification, and facilitate the generation of adenosine triphosphate for the growth and maintenance of dental biofilms [44,88]. L-arg has been considered an alkaline substrate, and certain species such as *S. gordonii*, *Streptococcus parasanguinis*, *Streptococcus intermedius*, *Streptococcus australis*, and *S. cristatus* contain ADS and catabolize it, promoting the alkalinity of the biofilm and inhibiting *S. mutans* [89,90,91]. In addition to affecting the change in the medium’s pH, L-arg can reduce adherence to the substrate and prevent bacterial congregation between *Prevotella oris* and *P. gingivalis*, inhibiting the growth of species such as *S. mutans*, *S. sobrinus*, and *S. sanguinis* while not affecting other bacteria. Promoting a change in the microbial community is therefore an alternative to favoring alkaline-base-producing bacteria and preventing the progression of caries [92,93].

Other compounds that have shown efficacy as prebiotics in other areas of the human body are currently being evaluated as prebiotics in the mouth. Substances such as glucomannan hydrolysate increase the growth of *Lactobacillus acidophilus*, which has probiotic properties and reduces the growth of *S. mutans* [94,95]. Similarly, lactitol, known as a prebiotic for gastrointestinal health, was evaluated by Slomka V. et al. [96], showing a specific increase in the growth of *S. salivarius* and no growth of pathogenic bacteria.

Sugar alcohols, such as arabinose, xylose, maltitol, and xylitol, have also been proposed as prebiotics due to their ability to act on acid production by saccharolytic bacteria, thereby reducing the risk of caries [78,97,98,99,100,101]. Kojima Y et al. [102] observed a favored growth of lactobacilli, which inhibited the growth of pathogenic species such as *Candida albicans*, *S. mutans*, and *P. gingivalis*.

The amino acid proline has also been postulated as a prebiotic. In vitro assays have indicated that proline affects host–pathogen interactions by modulating cell signaling and osmotic stress either as an antibacterial molecule or as a prebiotic [9,103]. Studies have also reported that a proline-containing peptide (tripeptide Ile-Pro-Ile (described as diprotin A)) led to increased resistance in biofilms to sucrose-induced decreases in pH [95,96]. Furthermore, the behavior of methionine--proline dipeptide has been evaluated, and it has been observed that methionine-proline succeeded in changing the composition of an oral biofilm model by reducing the pathogenic species to a predominantly beneficial species [104].

### 4.3. Preventive Approaches Related to Bacterial Coaggregation and Communication 

#### 4.3.1. Probiotics and Supragingival Homeostasis

Probiotics in the oral cavity are considered microorganisms that benefit oral health and have the potential to intervene in biofilm formation and in the niche’s pH among other actions, rebalancing the dysbiosis [102,105,106,107] but also producing effects on the host’s general health. Probiotics in the oral cavity act through three important mechanisms: direct bacterial inhibition through the production of antimicrobial substances, competition for nutrients and binding sites on host cell surfaces that prevent coaggregation, and modulation of the humoral and cellular immune response [107]. 

In caries prevention, probiotics can colonize the oral cavity and displace cariogenic bacteria. In general, probiotics can produce various antimicrobial substances—such as bacteriocins and bacteriocin-like peptides, lactic acid, and H_2_O_2_—that increase the medium’s pH and activate immune response cells such as macrophages, neutrophils, and natural killer cells to attack pathogens [107,108,109,110,111,112]. 

Several bacterial species of extraoral and intraoral origin have been proposed as oral probiotics, classified according to genus, species, and strain. Several species of the genus *Streptococcus* can attenuate the inflammatory response by decreasing interleukin-8 production by oral keratinocytes when attacked by *A. actinomycetemcomitans* [111,112]. The probiotics based on *S. cristatus* and *Streptococcus* A12 species produce proteases that interrupt bacteriocin production in *S. mutans* and produce antibacterial factors, such as organic acids and H_2_O_2_, that prevent the production of pathogenic bacterial compounds [42,48,108]. Other species, such as *S. gordonii* and *S. sanguinis*, produce H_2_O_2_, which inhibits the secretion of bacteriocins and interferes with intracellular signaling pathways to combat *S. mutans* [36,108,113]. The study by Thurnheer et al. [114] showed that *Streptococcus* produces bacteriocins that kill cryogenic bacteria and dampen salivary pH, preventing tissue demineralization. Similarly, the *S. salivarius* strain produces bacteriocins, such as streptin and salivaricin, with inhibitory power over other microorganisms. *S. salivarius* M18 produces urease and dextranase enzymes that neutralize the acidity of saliva and inhibit the microorganisms present in oral biofilms. *Streptococcus oligofermentans* has shown strong adhesion and a low capacity to metabolize carbohydrates but can produce H_2_O_2_ to inhibit pathogens such as *S. mutans* [115,116].

Various species of the genus *Lactobacillus*—including *Lactobacillus reuteri*, *Lactobacillus brevis*, *Lactobacillus rhamnosus*GG, *Lacticaseibacillus casei*, *Lactobacillus plantarum*, and *L. lactis*—have been reported to produce inhibitory substances such as ammonia that buffer the pH in saliva to prevent the growth of *S. mutans* [107,109,117]. Furthermore, as already mentioned, *L. lactis* produces nisin, a natural antimicrobial peptide highly active against Gram-positive bacteria with a potential role as a caries-preventive agent in the oral cavity with an effect on cariogenic bacteria and root canal pathogens. Nisin produce inhibition of common cariogenic-relevant bacteria such as *S. sanguinis*, *Lactobacillus fermenti*, *L. acidophilus* and *S. mutans*, which showed significant cell damage after nisin treatment. This confers considerable potential for used as an antibacterial agent to prevent dental caries and makes *L. lactis* a promising probiotic strain [56,57,58].

Other genera and species—such as *Neisseria*, *Streptococcus* sp. A12, *S. sanguinis* BCC23, *Actinomyces*, *Veillonella*, *Granulicatella*, diverse species of *Bifidobacterium* (*animalis*, *lactis*, *bifidum*, *longum*), and *Bacillus coagulans*—are currently being analyzed for their relationship to the recovery of symbiosis in biofilms [105,107,118,119,120].

Studies on the use of probiotics in the oral cavity are ongoing, and probiotics have demonstrated a beneficial role in preserving health; however, their effects are transitory, and strategies such as the use of prebiotics that enhance probiotics’ therapeutic effects are currently being researched [102,107,119].

Due to the rapid elimination of probiotic bacteria from the oral mucosa, the use of mucoadhesive and prebiotic polymer composites has been proposed as a strategy to prolong the contact time between the bacteria and the oral mucosa without delaying disintegration. One of these polymers that facilitates the prolongation and stability of probiotics in the mucosa is nanochitosan [121,122], which has been used to release salivary proteins and electrolytes, prolong the permanence of probiotics and therefore prebiotics in the mucosa, improve the pH of the medium, and prevent oral diseases [122,123]. Studies have shown that nanochitosan accompanied by sodium alginate and used as a coating for quercetin starch prolongs the resistance of *L. acidophilus*, *L. rhamnosus* GG, and other species such as *B. longum* [121,122,123,124,125,126]. 

#### 4.3.2. QS System Inhibition

The development and maturation of biofilms is regulated by various chemical communication systems between bacteria, known as the QS. When bacteria receive stimuli, the QS regulates gene expression by secreting specific signaling molecules, called autoinducers, which favor the development and communication of bacterial communities and incite coordinated behavior in the population. This mechanism also induces changes in the surface and in bacterial metabolism, which help form the biofilm matrix from the extracellular polysaccharide substances secreted by the bacteria [30,127,128].

The three most common autoinducer molecules are autoinducing peptides (AIP) produced by Gram-positive bacteria: autoinducer-2 (AI-2), present in Gram-positive and Gram-negative bacteria, and acyl homolactones (AHL), produced mainly by Gram-negative bacteria. A better understanding of the functioning of QS in dysbiotic processes has allowed the development of new strategies for controlling pathogen growth in oral biofilms. QS inhibition by degradation or inhibition of the synthesis of the signaling molecule IAs and interference of its functions in biological treatment systems are being considered as strategies. The use of a bacterial cell extract with QS enzymes that act extracellularly to degrade autoinducer molecules can be used to inhibit IA-2 and prevent biofilm formation in *S. mutans* [129,130,131]. Lactonase, acylase, decarboxylase, and deaminase have the ability to degrade AHL in biofilm formation [132]. D-galactose has been shown to reduce biofilm formation in *F. nucleatum*, *P. gingivalis*, and *T. forsythia* by blockading the AI-2 receptor [131,132,133]. AIP can affect intercellular communication and biofilm formation and promote microbial resistance. In the Streptococcus genus, a competence-stimulating peptide (CSP) has been identified that corresponds to AIP signaling. CSP-mediated QS is believed to inhibit and control mutacin (bacteriocin) transcription in *S. mutans*. When synthesized by commensal bacteria such as *Streptococcus oralis* and *A. naeslundii*, AI-2 allows beneficial growth among bacteria. However, AI-2 is mostly produced by pathogens such as *F. nucleatum*, *P. gingivalis*, and *Prevotella intermedia*, delaying the growth of commensal bacteria. AI-2 would therefore favor the change from a commensal to a pathogenic biofilm community. The role of AHL in oral biofilm formation has recently been demonstrated. This autoinducer has been identified in samples of saliva, tongue cells, and oral cavity biofilms containing isolated strains including *Enterobacter* sp., *Klebsiella pneumoniae*, *Pseudomonas putida*, and *Citrobacter amalonaticus* [129,132,133]. 

Although these new approaches have shown an ability to inhibit QS, their exact role and the mechanisms of QS inhibition have not been precisely defined, and further studies are required [129].

#### 4.3.3. Glucose Oxidase Nanohybrid (Dex-IONP-Gox)

A bifunctional nanohybrid system for selectively targeting pathogenic bacteria and preserving commensal bacteria in oral biofilms has recently been described. The system is composed of a glucose oxidase (GOx) that is covalently attached to a dextran-coated iron oxide nanoparticle (Dex-IONP). GOx can catalyze intracellular glucose and oxygen to increase H_2_O_2_ in the biofilm, while Dex-IONP decomposes H_2_O_2_ into oxygen ions, free radicals that induce reactive oxygen species with capacity for inhibit bacteria and degrade the extracellular polysaccharide substance matrix [134]. The study by Yue Huang et al. [134] showed that Dex-IONP-GOx preferentially binds and kills *S. mutans* more effectively than commensal *S. oralis*, an important strategy given that it does not disturb the commensals and host microbiota. The strategy also showed efficacy in reducing caries when compared with chlorhexidine without producing side effects on tissues and without affecting the gastrointestinal microbiome.

## 5. Conclusions

Dental caries is a highly prevalent multifactorial disease that results from the interaction between cariogenic bacteria, a diet rich in fermentable carbohydrates, and a susceptible host. Dental caries is mainly combated with daily oral hygiene through various strategies, which even today are not entirely effective. Undesirable effects such as pigmentation and bacterial resistance have therefore increased. There is therefore a need for new therapeutic pathways that focus on preserving the balance in the oral microbiota, applying strategies to combat caries while maintaining biofilm integrity and homeostasis in the rapid phase of supragingival plaque formation from bacteria firmly adhered to a previously clean surface.

Long-term prevention will only be achieved if oral homeostasis is maintained by controlling the factors that favor dysbiosis. If these mechanisms are not addressed, the disease might reappear. This literature review discussed the general benefits of these strategies and their mechanisms of action in the fight against oral pathogenic bacteria in the early stages of biofilm formation after the daily hygiene process. More studies are needed to better understand the mechanisms of action, toxicity, and effectiveness of these strategies with the aim of developing commercial products that are easily accessible to the entire population and thus mitigate the high rates of tooth loss due to caries and improve quality of life.

## Data Availability

Not applicable.
